# Development and validation of machine learning models for MASLD: based on multiple potential screening indicators

**DOI:** 10.3389/fendo.2024.1449064

**Published:** 2025-01-21

**Authors:** Hao Chen, Jingjing Zhang, Xueqin Chen, Ling Luo, Wenjiao Dong, Yongjie Wang, Jiyu Zhou, Canjin Chen, Wenhao Wang, Wenbin Zhang, Zhiyi Zhang, Yongguang Cai, Danli Kong, Yuanlin Ding

**Affiliations:** ^1^ Department of Epidemiology and Medical Statistics School of Public Health, Guangdong Medical University, Dongguan, Guangdong, China; ^2^ Department of Medical Oncology, Central Hospital of Guangdong Nongken, Zhanjiang, Guangdong, China

**Keywords:** metabolic dysfunction-associated steatotic liver disease, machine learning, insulin resistance, triglyceride glucose, risk prediction model

## Abstract

**Background:**

Multifaceted factors play a crucial role in the prevention and treatment of metabolic dysfunction-associated steatotic liver disease (MASLD). This study aimed to utilize multifaceted indicators to construct MASLD risk prediction machine learning models and explore the core factors within these models.

**Methods:**

MASLD risk prediction models were constructed based on seven machine learning algorithms using all variables, insulin-related variables, demographic characteristics variables, and other indicators, respectively. Subsequently, the partial dependence plot(PDP) method and SHapley Additive exPlanations (SHAP) were utilized to explain the roles of important variables in the model to filter out the optimal indicators for constructing the MASLD risk model.

**Results:**

Ranking the feature importance of the Random Forest (RF) model and eXtreme Gradient Boosting (XGBoost) model constructed using all variables found that both homeostasis model assessment of insulin resistance (HOMA-IR) and triglyceride glucose-waist circumference (TyG-WC) were the first and second most important variables. The MASLD risk prediction model constructed using the variables with top 10 importance was superior to the previous model. The PDP and SHAP methods were further utilized to screen the best indicators (including HOMA-IR, TyG-WC, age, aspartate aminotransferase (AST), and ethnicity) for constructing the model, and the mean area under the curve value of the models was 0.960.

**Conclusions:**

HOMA-IR and TyG-WC are core factors in predicting MASLD risk. Ultimately, our study constructed the optimal MASLD risk prediction model using HOMA-IR, TyG-WC, age, AST, and ethnicity.

## Introduction

In June 2023, the international consensus group introduced the term “ Steatotic Liver Disease (SLD)” as an inclusive term covering all different etiologies of hepatic steatosis, including metabolic, alcohol-related, drug-induced, and cryptogenic causes (1). Metabolic dysfunction-associated steatotic liver disease (MASLD), previously known as Non-alcoholic fatty liver disease (NAFLD), is one of the most common chronic liver diseases worldwide, affecting approximately 30% of the global population (2). MASLD typically progresses over time, potentially leading to hepatic inflammation (metabolic dysfunction-associated steatohepatitis, MASH), liver fibrosis, and ultimately, the development of cirrhosis or even hepatocellular carcinoma (3). With the continued increase in obesity and diabetes mellitus (DM), the prevalence of NAFLD and associated healthcare costs are expected to rise, significantly impacting global public health. MASLD is also considered a hepatic manifestation of metabolic syndrome, as it is closely associated with metabolic disorders such as obesity, dyslipidemia, and DM. Early screening and effective intervention measures help reduce and delay the occurrence of adverse prognostic events associated with MASLD. Liver biopsy has long been considered the gold standard for histological assessment, diagnosis, and prognosis determination of liver fibrosis (4). However, its invasive nature, potential risk of bleeding, and sampling errors due to uneven distribution of liver parenchymal lesions make it difficult to be widely used in clinical practice (5), resulting in a large number of MASLD patients missing the optimal timing for diagnosis and treatment. Therefore, exploring accurate and non-invasive biomarkers for the diagnosis of MASLD is crucial to reduce the need for invasive liver biopsy and identify patients at high risk of liver and metabolic complications early on.

Machine learning (ML) is a branch of artificial intelligence with the capability to handle large, complex, and entirely different datasets, creating complex analytical models based on learning frameworks, thereby improving and optimizing prediction accuracy (6). Therefore, an increasing number of researchers are beginning to develop disease risk prediction models through ML (7–11). Numerous studies have utilized clinical data and machine learning algorithms to construct NAFLD risk prediction models. Zhou et al. (11) developed a NAFLD risk prediction model based on obese children, which demonstrated good clinical discriminative ability, with an area under the curve (AUC) value of 0.821 for the receiver operating characteristic (ROC) curve. Liu et al. (12) constructed a NAFLD risk prediction model based on a population undergoing health checkups, using machine learning algorithms. eXtreme Gradient Boosting (XGBoost) demonstrated excellent clinical predictive value, with an AUC value of 0.926 for the ROC curve. Huang et al. (13) developed a risk prediction model for NAFLD within a population based on prospective cohort studies, utilizing various machine learning algorithms. The best model, Categorical Boost (CatBoost) achieved a predictive performance of AUC = 0.810.

However, available MASLD risk prediction models are scarce currently, and previous studies have seldom explored the roles of various indicators in NAFLD risk prediction models, making it difficult to assess which indicators play a core role in predicting MASLD risk. Therefore, in this study, we aimed to utilize multifaceted indicators from the National Health and Nutrition Examination Survey (NHANES) data from 2005-2010 and 2015-2018 to construct MASLD risk prediction machine learning models and explore the core factors within the models.

## Materials and methods

### Study design and population

**Figure 1:**
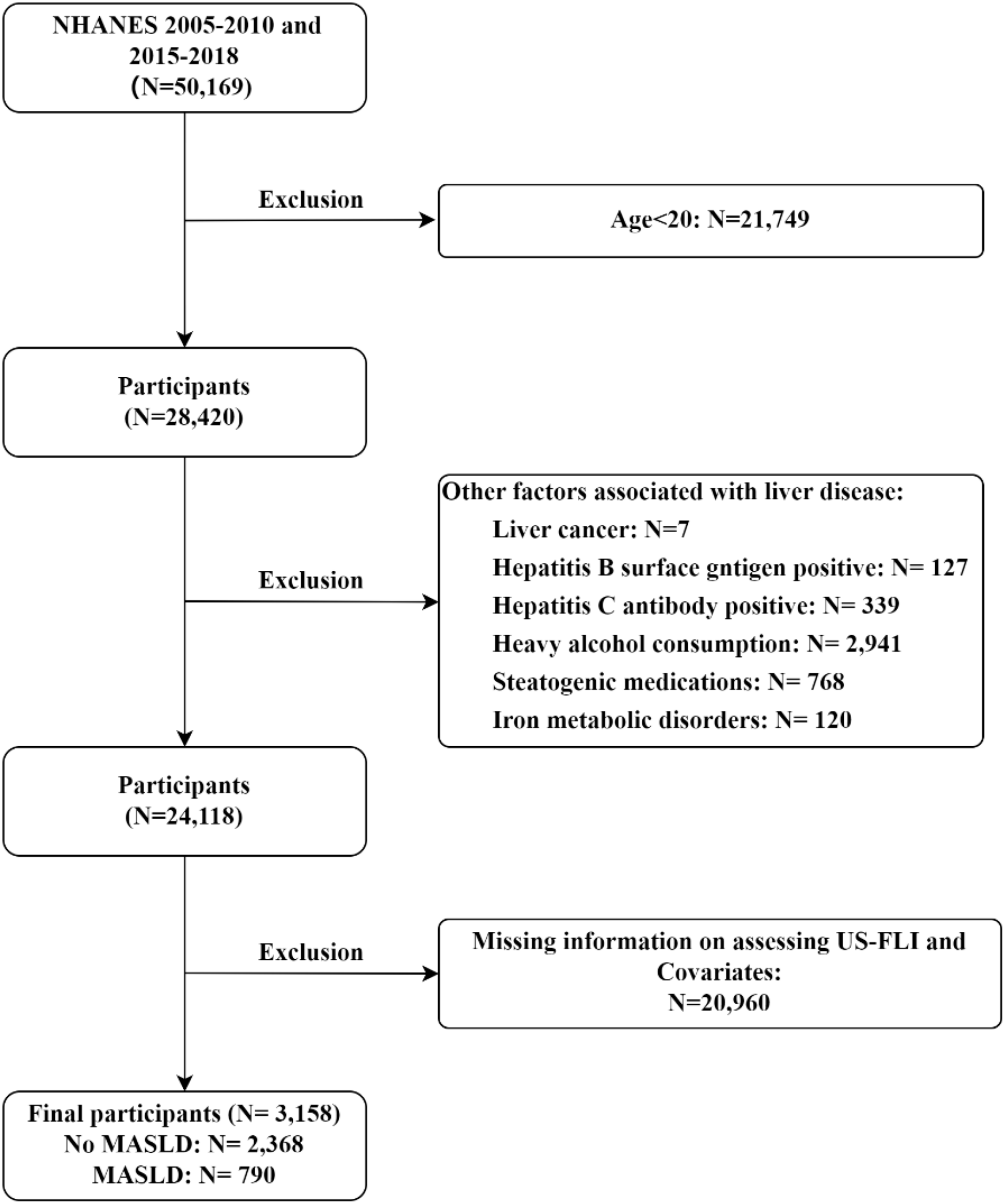
Flow chart of subject inclusion and exclusion in the 2005-2010, 2015-2018 U.S. National Health and Nutrition Examination Survey.

Study data is from NHANES and includes 5 cycles from 2005-2010 and 2015-2018. The exclusion criteria adopted in this study were as follows (1) participants younger than 20 years of age; (2) participants with liver disease caused by other factors, including 1) iron metabolic disorders, indicated by ferritin concentration exceeding 200ug/L; 2) alcohol-related liver disease, characterized by heavy drinking (≥3 drinks per day for females and ≥4 drinks per day for males) or binge drinking (≥5 drinks on a single occasion); 3) hepatitis virus infection, identified by the presence of hepatitis B surface antigen or hepatitis C confirmation antibody; 4) self-reported liver cancer; 5) taking steatogenic medications for at least 6 months(including amiodarone, methotrexate, tamoxifen, aspirin, ibuprofen, nucleoside reverse transcriptase inhibitors, protease inhibitors, valproic acid, carbamazepine, glucocorticoids); (3) participants who lacked the information used to assess MASLD; and (4) participants who were missing other covariates. The detailed flowchart is shown in **Figure 1**. 3,158 participants were finally included, consisting of 2,368 non-MASLD patients and 790 MASLD patients.

### Definition of MASLD

The diagnosis of MAFLD typically relies on techniques such as abdominal ultrasonography, magnetic resonance imaging, and other imaging modalities aimed at identifying liver fat accumulation. Further confirmation may necessitate a liver biopsy. However, the latter is not commonly employed due to its high operator dependence, cost considerations, and the requirement for significant steatosis, typically exceeding 20-30% of liver cells, for detection. Consequently, alternative approaches have been developed to address these limitations. One such method is the United States Fatty Liver Index (US-FLI), pioneered by CERuhl, designed specifically for assessing fatty liver disease within the U.S. population (14). The specific calculation formula for the United States fatty liver index (US-FLI) was as follows:


$$US-FLI=\frac{e^{-0.8073*non-Hispanic\;black+0.3458*Mexican\;American+0.0093*age+0.6151*\log_{e}(GGT)+0.0249*waist\;circumference+1.1792*\log_{e}(insulin)+0.8242*\log_{e}(glu\cos e)-14.7812}}{1+e^{-0.8073*non-Hispanic\;black+0.3458*Mexican\;American+0.0093*age+0.6151*\log_{e}(GGT)+0.0249*waist\;circumference+1.1792*\log_{e}(insulin)+0.8242*\log_{e}(glu\cos e)-14.7812}}*100$$

In the exclusion of other liver diseases associated with the aforementioned factors, when US-FLI≥30, we considered the participant to have MASLD.

### Study covariates

In this study, we considered several covariates that could potentially confound the outcomes: 1) Demographic variables: age, gender, ethnicity, education level, military status, marital status, sleep status, smoke status, the family income poverty ratio (PIR), physical activity (PA); 2) Examination variables: waist circumference (WC), waist to height ratio (WtHR), and body mass index (BMI); 3) Laboratory variables: low-density lipoprotein cholesterol (LDL-C), high-density lipoprotein cholesterol (HDL-C), total cholesterol (TC), triglyceride (TG), alanine transaminase (ALT), aspartate aminotransferase (AST), bilirubin, albumin, fasting blood glucose (FBG), triglyceride glucose (TyG) index–related parameters, including the TyG, TyG–BMI, TyG-WtHR, and TyG-WC, Homeostasis Model Assessment of Insulin Resistance (HOMA-IR); 4) Medical history: diabetes mellitus (DM), hypertension, cardiovascular disease (CVD); 5) Other indexes: dietary inflammatory index (DII), systemic immune-inflammation index (SII), oxidative balance score (OBS), composite dietary antioxidant index (CDAI), visceral adiposity index (VAI), lipid accumulation product (LAP).

### Statistical analyses

In this study, data were analyzed and visualized using R 4.0.1 software. Continuous variables were described using mean ± standard deviation (SD) or median (IQR), while categorical variables were expressed as percentages. The Shapiro-Wilk test was employed to assess the normality of continuous variables, and the independent sample t-test or Mann-Whitney test was used for between-group comparisons of continuous variables. Between-group comparisons of categorical variables were conducted using the chi-square test or Fisher’s exact test.

### Construction of the machine learning model

Research data was split between 70% as training sets (n = 2,211) and 30% as testing sets (n =947).

In this study, seven different machine learning algorithms, namely Random Forest (RF), Support Vector Machine (SVM), k-Nearest Neighbor (KNN), eXtreme Gradient Boosting (XGBoost), Naïve Bayes Model (NBM), Backpropagation Neural Network (BPNN), and Logistic Regression (LG), were used to evaluate the predictive effectiveness of various variable characteristics on MASLD, each with its own features. RF can handle high-dimensional data and has a strong resilience to noise. It is robust against overfitting when hyperparameters are optimized, but has high time complexity with large datasets (15). SVM is effective for both linear and non-linear classification tasks and performs well on high-dimensional datasets. It is less affected by outliers, but its time complexity also can be high when working with large sample sizes (16, 17). KNN is simple to implement and highly accurate, making it ideal for data without strict assumptions. It handles outliers well but can be computationally expensive and slow when the dataset is large (18, 19). Known for its high accuracy and efficiency, XGBoost is well-suited for complex, high-dimensional datasets. It includes options for regularization to prevent overfitting, although it can be computationally intensive if not properly tuned (20). NBM is efficient and works well with small datasets, especially when the assumption of feature independence is approximately met. However, it may perform poorly with data where features are heavily correlated or when feature distribution assumptions are violated (21, 22). BPNN is powerful for capturing complex patterns in large datasets and is capable of handling non-linear relationships. However, it requires significant computational resources and training time, and it is prone to overfitting without proper regularization or dropout methods (23–25). LR is interpretable and effective for binary classification tasks with linear relationships. It has low time complexity, making it efficient for large datasets, but it may underperform with non-linear or complex patterns (26, 27). To evaluate the predictive effect of various types of variables on MASLD, we constructed models utilizing 1) all variables; 2) insulin-related indexes (HOMA-IR, TyG index-related parameters); 3) demographic characteristics variables; and 4) other indexes, respectively. Subsequently, to develop a more accurate and parsimonious risk prediction model, we selected the feature variables from the best-performing model among the four evaluated models, ranking them by importance. The top 10 variables were chosen based on their importance rankings and used to construct a refined MASLD risk prediction model. To avoid overfitting the models, we performed hyperparameter optimization for each model. In addition, considering the robustness and generalizability of the models, we integrated multiple evaluation metrics of seven machine learning algorithms and performed 10-fold cross-validation for each model.

### Interpretable methods pipeline of prediction models

We applied interpretability techniques, specifically Partial Dependence Plots (PDPs) and SHapley Additive exPlanations (SHAP), to better understand each variable’s contribution to MASLD risk prediction. PDPs were used to illustrate the marginal effect of each individual variable on MASLD risk by showing how the predicted risk changes across a range of values for each variable while keeping other features constant. This helps isolate the impact of each feature on the outcome and provides a clearer picture of its direction and strength in influencing MASLD risk.

In addition, SHAP was employed to quantify and visualize the contributions of each variable across different predictions, offering insights into feature importance and interaction effects. SHAP values reveal how much each variable pushes the prediction toward or away from higher MASLD risk in individual cases. By integrating insights from both PDP and SHAP analyses, we identified the most influential features and refined our model to retain only those with substantive predictive power, thereby constructing an optimized final model.

### Evaluation of machine learning model

The performance of the model was assessed by various evaluation metrics including receiver operating characteristic curve (ROC), area under the receiver operator curve (AUC), accuracy, sensitivity/recall, specificity, false positive rate (FPR), false negative rate (FNR), positive predictive value (PPV), negative predictive value (NPV) and the F_1_ score. The AUC was mainly used as an evaluation metric for performance comparison between models. The variance inflation factor (VIF) was used to evaluate multicollinearity in the multivariate analysis based on the intercorrelation between variables.

## Results

### Characteristics of the study population

**Table 1** displays the general characteristics of the study participants. A total of 3,158 U.S. adults were included in the study, with a greater proportion of MASLD patients being male, non-Hispanic whites. Hypertension, DM, and CVD may all be risk factors for MASLD. In addition, compared with participants without MASLD, participants with MASLD have higher levels of HOMA-IR, TyG index-related parameters, DII, SII, VAI, and LAP index relative to participants without MASLD.

**Table 1:** Baseline characteristics of participants.

	No MASLD (N=2,368)	MASLD (N=790)	*P*
Age (mean ± SD)	48.79 ± 17.13	55.41 ± 15.50	<0.001
PIR (mean ± SD)	2.94 ± 1.63	2.75 ± 1.64	<0.05
TC (mean ± SD)	5.08 ± 1.03	5.08 ± 1.06	0.995
HDL (mean ± SD)	1.49 ± 0.41	1.20 ± 0.30	<0.001
LDL (mean ± SD)	3.04 ± 0.91	3.06 ± 0.94	0.651
TG (median, IQR)	1.05 (0.73)	1.61 (1.11)	<0.001
BMI (mean ± SD)	26.73 ± 5.26	33.33 ± 5.94	<0.001
WC (mean ± SD)	93.06 ± 12.96	111.66 ± 13.20	<0.001
FBG (median, IQR)	5.38 (0.72)	6.00 (1.22)	<0.001
ALT (median, IQR)	20.00 (10.00)	26.00 (14.00)	<0.001
AST (median, IQR)	23.00 (7.00)	24.00 (8.00)	<0.001
Bilirubin (mean ± SD)	13.77 ± 5.19	13.76 ± 5.18	0.951
Albumin (mean ± SD)	42.45 ± 3.07	41.88 ± 2.92	<0.001
PA (median, IQR)	1,280.00 (3300.00)	1250.00 (2980.50)	0.173
TyG (mean ± SD)	8.45 ± 0.55	9.03 ± 0.52	<0.001
HOMA-IR (median, IQR)	1.78 (1.41)	5.05 (3.61)	<0.001
WtHR (mean ± SD)	0.55 ± 0.08	0.66 ± 0.08	<0.001
TyG-BMI (mean ± SD)	226.59 ± 49.97	300.77 ± 55.96	<0.001
TyG-WC (mean ± SD)	788.83 ± 135.39	1,008.07 ± 134.32	<0.001
TyG-WtHR (mean ± SD)	4.69 ± 0.81	5.95 ± 0.79	<0.001
DII (mean ± SD)	1.33 ± 1.90	1.51 ± 1.80	<0.05
SII (mean ± SD)	519.20 ± 307.72	561.82 ± 295.45	0.001
OBS (mean ± SD)	20.08 ± 8.60	18.84 ± 8.06	<0.001
CDAI (mean ± SD)	0.82 ± 4.08	0.52 ± 3.76	0.061
VAI (median, IQR)	1.16 (1.11)	2.25 (1.93)	<0.001
LAP (median, IQR)	32.58 (18.50)	77.42 (54.69)	<0.001
Gender (N, %)			<0.001
Female	1,222 (51.6)	287 (36.3)	
Male	1,146 (48.4)	503 (63.7)	
Ethnicity (N, %)			<0.001
Non-Hispanic White	1,233 (52.1)	436 (55.2)	
Non-Hispanic Blacks	534 (22.6)	86 (10.9)	
Mexican	270 (11.4)	166 (21.0)	
Other	331 (14.0)	102 (12.9)	
Education (N, %)			<0.001
Elementary and secondary education	429 (18.1)	202 (25.6)	
High school	539 (22.8)	193 (24.4)	
Bachelor degree or higher	1,400 (59.1)	395 (49.9)	
Military (N, %)			<0.001
No	2,073 (87.5)	632 (80.0)	
Yes	295 (12.5)	158 (20.0)	
Marital (N, %)			<0.001
Married or Living with partner	1,508 (63.7)	552 (69.9)	
Widowed, Divorced, or Separated	476 (20.1)	170 (21.5)	
Never married	384 (16.2)	68 (8.6)	
Sleep status (N, %)			0.473
Short	898 (37.9)	311 (39.4)	
Normal	1,305 (55.1)	433 (54.7)	
Long	165 (7.0)	46 (5.8)	
Smoke status (N, %)			<0.001
Never	1,385 (58.5)	421 (53.3)	
Former	583 (24.6)	267 (33.8)	
Now	400 (16.9)	102 (12.9)	
Hypertension (N, %)			<0.001
No	1,569 (66.3)	321 (40.6)	
Yes	799 (33.7)	469 (59.4)	
DM (N, %)			<0.001
No	2,087 (88.1)	530 (67.1)	
Yes	281 (11.9)	260 (32.9)	
CVD (N, %)			<0.001
No	2,208 (93.2)	671 (84.9)	
Yes	160 (6.8)	119 (15.1)	

### Evaluation and comparison of the predictive models

**Figure 2:**
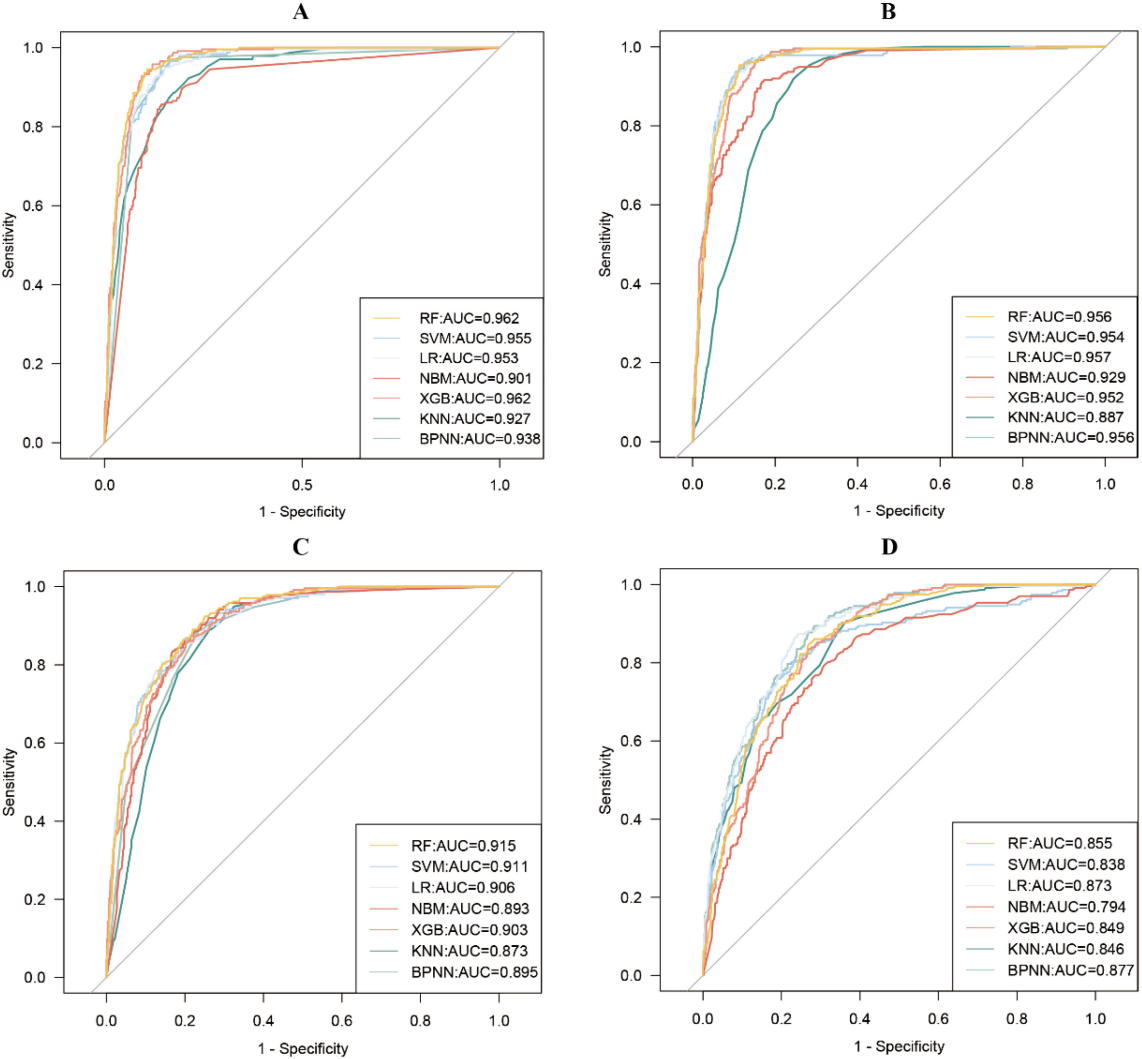
Receiver Operating Characteristic (ROC) curves for seven machine learning models are constructed using different variables. **(A)** all variables; **(B)** insulin-related indexes; **(C)** demographic characteristics variables; **(D)** other indexes.

**Figure 2** depicts the ROC curves for each of the seven models constructed using different variables. The results of other evaluation metrics of the models can be viewed in **Supplementary Tables S1-S4**. In the test set, the overall effect of the models constructed using insulin-related indexes was slightly better than that of the models constructed using all variables, but the effect of the overall ROC curve of the seven models constructed using all variables (average AUC: 0.942) was slightly higher than that of the models constructed using only insulin-related indexes (average AUC: 0.941). In addition, in the analysis of multicollinearity between the two models, it was found that there was multicollinearity between several variables in both models (VIF>10).

### Predictive models constructed with top 10 variables

**Figure 3:**
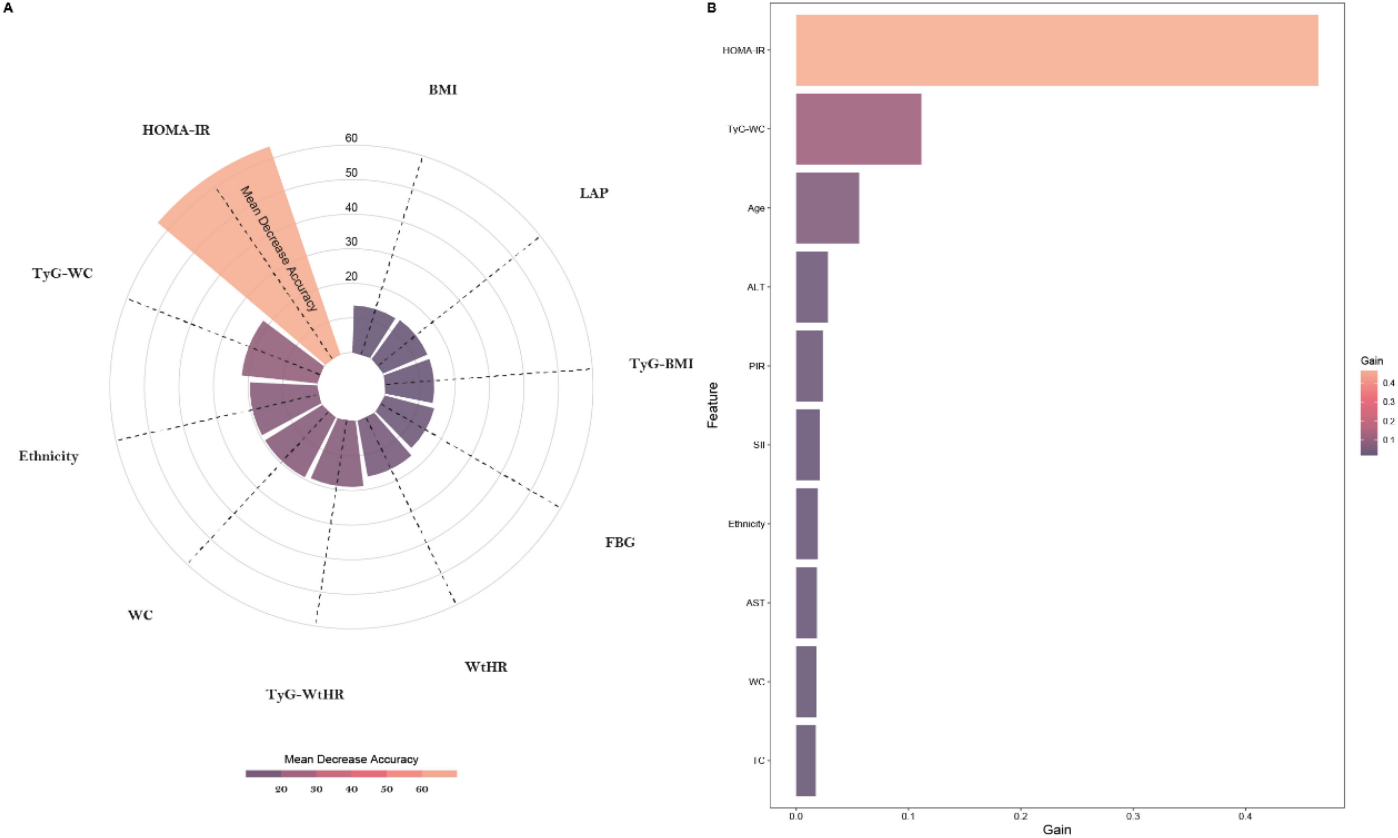
The contribution of the top 10 variables in predictive models. **(A)** The contribution of the top 10 variables in the RF predictive model; **(B)** The contribution of the top 10 variables in the XGBoost predictive model.

To further explore the optimal solution for constructing the MASLD risk prediction model, we selected the two predictive models (RF and XGBoost) with the highest AUC values among the seven models constructed with all variables and filtered out the top 10 importance variables in the two predictive models. **Figure 3** illustrates the top 10 variables of importance for the two models. Focusing on the top 10 variables, which contribute most significantly to model output, allows us to provide clearer insights into the key factors influencing MASLD risk without overwhelming the interpretation with too many variables.

**Figure 4:**
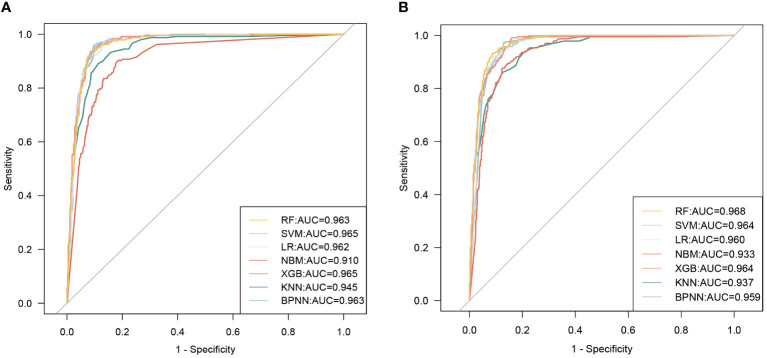
ROC curves for seven machine learning models are constructed using the top 10 variables of importance. **(A)** model uses the top 10 variables of importance in the RF model; **(B)** model uses the top 10 variables of importance in the XGBoost model.

Therefore, we constructed disease prediction models for MASLD using the top10 variables in importance in RF and XGBoost, respectively (Hereafter referred to as RF top10 models and XGBoost top10 models). It was found that the mean AUC values of the RF top 10 models and the XGBoost top 10 models did not differ much (slightly higher in the XGBoost top 10 models), but both were higher than the model constructed using only the insulin-related indexes. **Figure 4** illustrates the results of the ROC curves for the models, and the results for the other evaluation metrics are shown in **Supplementary Tables S5, S6**.

### Interpretation of ML models

**Figure 5:**
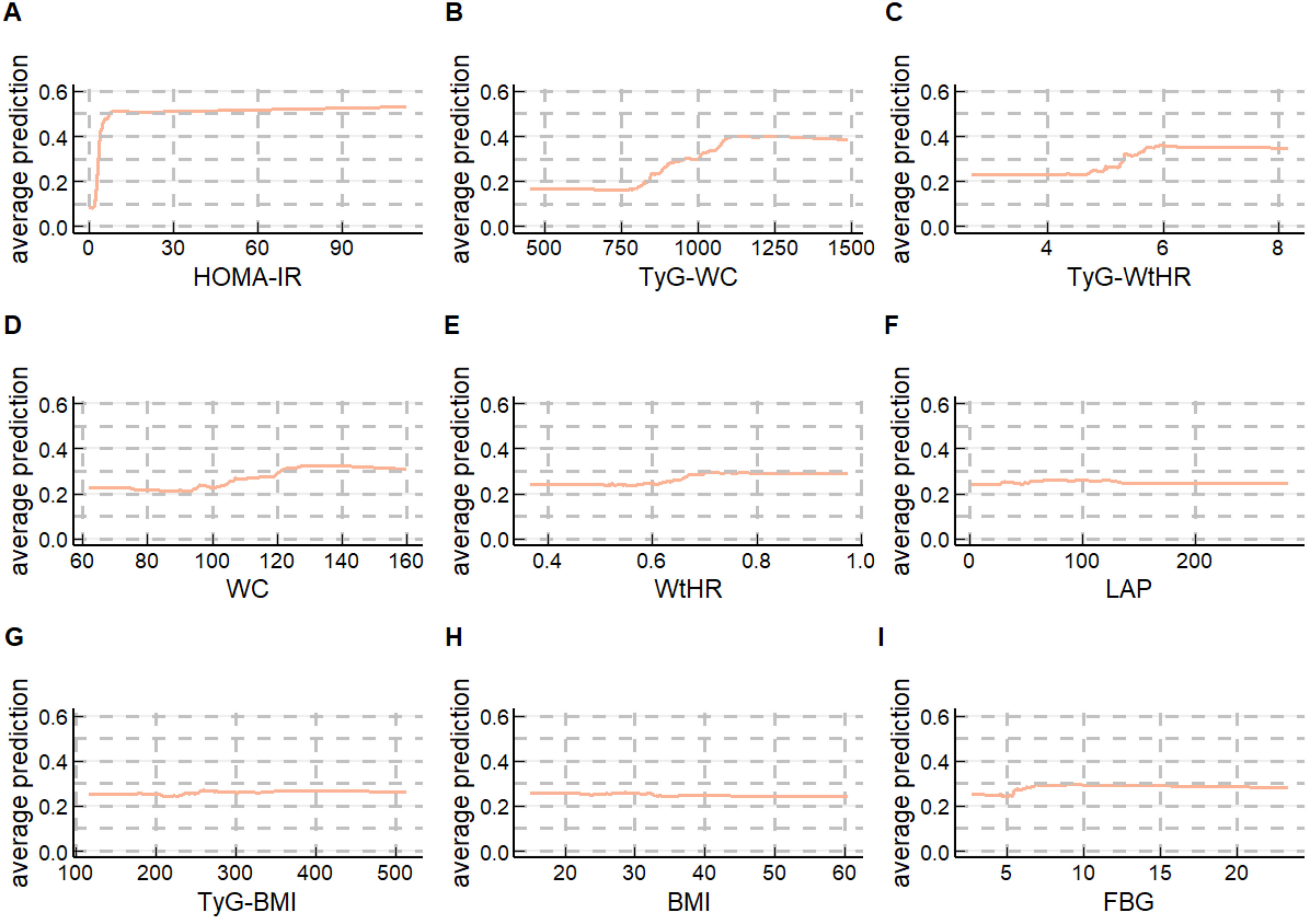
Partial dependence plots (PDPs) of the RF top 10 model **(A)** HOMA-IR’ PDP of the RF top 10 model; **(B)** TyG-WC’ PDP of the RF top 10 model; **(C)** TyG-WtHR’ PDP of the RF top 10 model; **(D)** WC’ PDP of the RF top 10 model; **(E)** WtHR’ PDP of the RF top 10 model; **(F)** LAP’ PDP of the RF top 10 model; **(G)** TyG-BMI’ PDP of the RF top 10 model; **(H)** BMI’ PDP of the RF top 10 model; (I)FBG’ PDP of the RF top 10 model.

**Figure 6:**
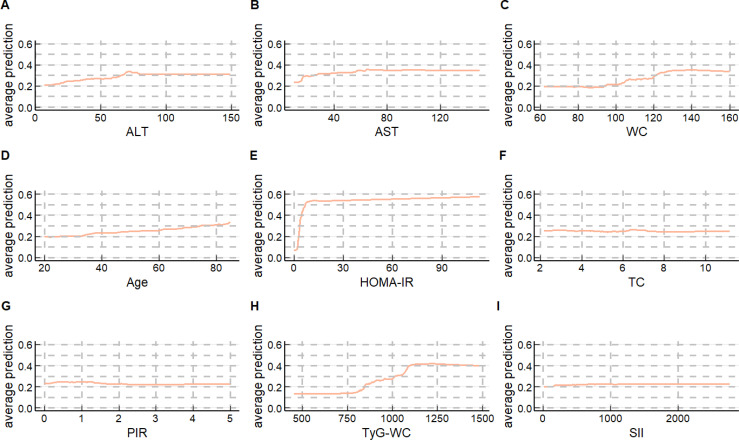
Partial dependence plots (PDPs) of the XGBoost top 10 model **(A)** ALT’ PDP of the XGBoost top 10 model; **(B)**) AST’ PDP of the XGBoost top 10 model; **(C)** WC’ PDP of the XGBoost top 10 model; **(D)** Age’ PDP of the XGBoost top 10 model; **(E)** HOMA-IR’ PDP of the XGBoost top 10 model; **(F)** TC’ PDP of the XGBoost top 10 model; **(G)** PIR’ PDP of the XGBoost top 10 model; **(H)** TyG-WC’ PDP of the XGBoost top 10 model; **(I)** SII’ PDP of the XGBoost top 10 model.

Since the difference in the overall predictive performance between the RF top 10 models and the XGBoost top 10 models is very small, to further explore the models, we chose to generate PDPs for the Random Forest model selected from both the RF top 10 models and the XGBoost top 10 models to interpret the predictive models. As presented in **Figures 5**, **6**, the application of PDPs allowed for a broader interpretation of model performance, which displayed the relationship between the features and MASLD. The PDP analysis of the RF top 10 models indicates that increases in nine continuous variables included in the model are associated with elevated risk predictions for MASLD. Specifically, HOMA-IR, TyG-WC, and TyG-WtHR exhibit the most significant effects. More specifically, as the levels of HOMA-IR range from approximately 0 to 10, TyG-WC range from approximately 818 to 1091, and TyG-WtHR range from approximately 5 to 6, the risk prediction values for MASLD show an increasing trend. The PDP analysis results of the XGBoost top 10 models are generally consistent with those of the RF top 10 models. It is noteworthy that, unlike HOMA-IR and TyG-WC, which only impact the risk prediction values for MASLD within specific ranges, the risk prediction values for MASLD increase with age across the entire age range.

PDPs can also provide insights into the interaction performance of a model. **Supplementary Figures S1, 3** show the overall interaction strength between variables in the two models, respectively. **Supplementary Figures S2, 4** display the synergistic effects of variables’ levels on MASLD risk. The results still indicate that HOMA-IR, TyG-WC, and TyG-WtHR dominate in predicting MASLD in the RF top 10 models; In the XGBoost top 10 models, the predicted values of MASLD are mainly determined by HOMA-IR, TyG-WC, and age.

Due to the limitations of PDPs, we could not assess the role of ethnicity in the model. Meanwhile, we observed that variables such as ALT and AST seem to play a role in predicting MASLD risk. To further explore the optimal model configuration, we conducted a SHAP analysis on the two models mentioned above (**Supplementary Figures S5**, **S6**). The results from the SHAP dependency plots indicate that SHAP values vary across different ethnic groups, suggesting that ethnicity might be a potential risk factor for MASLD and that the effect of TyG-WC may vary across ethnicities. From the color mapping in the SHAP plots, it can be observed that AST is not influenced by HOMA-IR in the model, while ALT is significantly influenced by TyG-WC. In the HOMA-IR plot, where color mapping represents Age, data points with lighter colors (representing older age) are distributed in regions with higher HOMA-IR values and SHAP values, indicating that HOMA-IR may have a greater impact on MASLD risk in older populations.

### Construction and evaluation of the optimal MASLD prediction model

**Figure 7:**
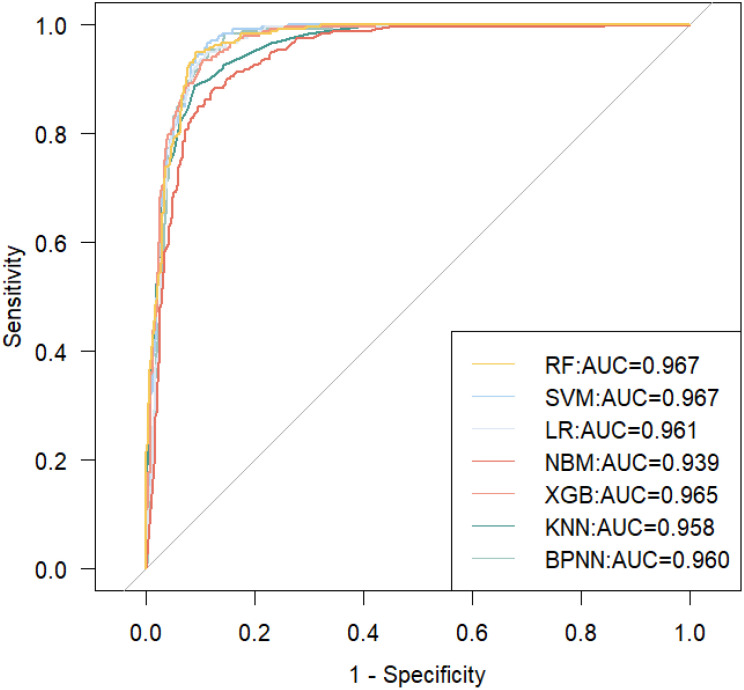
ROC curves for seven machine learning models are constructed using HOMA-IR, TyG-WC, age, AST, and ethnicity.

Taking into account the effects of the variables in both the PDPs and SHAP results on MASLD risk prediction performance as well as interactions and covariances, we further screened the variables used to construct the model. Eventually, we utilized 5 factors (including HOMA-IR, TyG-WC age, AST, and ethnicity) to construct the optimal MASLD risk prediction model (**Figure 7**, average AUC=0.960). We also calculated the VIF of all the variables in this model and proved that there is no multicollinearity between them (VIF<10).

## Discussion

MASLD, the most common chronic liver disease worldwide, is mediated by various factors including genetic susceptibility, dietary habits, obesity, insulin resistance, and the endocrine effects of many diseases. Consequently, extensive research is underway to explore non-invasive, practical, and reliable disease prediction models to identify and manage individuals at high risk of MASLD, ultimately alleviating the disease burden. In this large cross-sectional study, our primary aim is to investigate how to construct the optimal MASLD prediction model and the role played by indicators in the model. Despite NAFLD being eventually renamed as MASLD, studies have shown excellent consistency between the definitions of NAFLD and MASLD, with approximately 99% of NAFLD patients meeting MASLD criteria (28). Therefore, while this study focuses on MASLD, it also discusses NAFLD and MAFLD simultaneously.

Previous studies have already indicated that obesity is an independent risk factor for NAFLD (29). The new nomenclature and definition more intuitively reflect that metabolism (obesity and DM) is a key etiology of fatty liver disease. Our study results also found that the average BMI and HOMA-IR of MASLD patients were 33.33 kg/m2 and 5.06, respectively. Additionally, the proportion of participants with diabetes mellitus (DM) was much higher in MASLD patients compared to those without MASLD. Furthermore, our study found that MASLD patients had higher SII and DII compared to participants without MASLD, consistent with the findings of Yan et al. (30–33).

Inflammation plays a crucial role in the progression of MASLD to MASH, liver fibrosis, and even liver cancer (34). In addition to the direct effects of immune-inflammatory factors, these cytokines also promote the development of IR and T2DM through activation of intracellular pathways, thereby influencing MASLD (35). Studies have also found that higher levels of OBS have a protective effect on MASLD, and Tan et al. (36) suggested that OBS may influence MASLD by reducing insulin resistance levels. Previous studies consistently demonstrate a negative correlation between PA and NAFLD, suggesting that even mild exercise can prevent and treat NAFLD to some extent (37, 38). However, this study did not observe an association between PA and MASLD, speculating that most MASLD patients in the early stages of liver disease prefer to improve their disease burden by adjusting dietary habits. Additionally, MASLD patients are mostly obese and may be less inclined to choose increased physical activity as a means to improve their condition. VAI and LAP are also positively correlated with MASLD, consistent with the findings of previous studies by Vural et al (39, 40). Additionally, Peng et al. (41) suggested that LAP may have better predictive performance than VAI, a viewpoint supported by our results. However, studies by Boden et al. (42–44) suggest that increased visceral adipose tissue may be achieved through pathways such as oxidative stress, inflammation, and IR. Despite the multifactorial etiology of MASLD, IR, and obesity remain core driving factors in its development. Therefore, for research aiming to predict and assess MASLD risk, it is imperative to delve deeper into these core driving factors and explore their role in MASLD risk assessment through model construction.

HOMA-IR, as a commonly used index for measuring IR, has been widely applied. However, its requirement for fasting insulin levels to some extent limits its practicality in clinical settings. In contrast, the TyG index addresses this limitation, becoming a simple, reproducible, and reliable indicator for assessing IR. Previous studies have demonstrated that TyG-related indices have good predictive performance for MAFLD (45). Xue et al. (46) used logistic regression to explore the association between insulin-related indexes and MAFLD, evaluating the predictive performance of individual indicators on MAFLD. They found that TyG-WC had the best predictive performance for MAFLD (AUC=0.832). Similar results were obtained by Peng et al. (41). Therefore, this study integrated insulin-related indexes to construct models for predicting MASLD risk. The performance of the LR model (AUC=0.957) was far superior to the models constructed by Peng (41) and Xue (46) et al. using single indicators. This significant performance difference emphasizes the predictive capability of the composite index model. To further obtain indicators that more comprehensively reflect MASLD risk and construct the optimal MASLD risk prediction model, we selected the top 10 variables of importance in the RF model and XGBoost model constructed using all variables. We found that HOMA-IR and TyG-WC were ranked first and second, respectively, fully reflecting the importance of insulin resistance in MASLD. Additionally, ethnicity and WC also appeared in the top 10 of importance in both models. The genotypes and lifestyle habits of different ethnic groups may be major factors influencing MASLD. WC, as an important indicator of central obesity, can also reflect the risk of MASLD to some extent. However, its importance is much lower than that of TyG-WC, possibly because TyG-WC integrates indicators of IR and central obesity, making it more prominent in predicting MASLD risk.

We further constructed MASLD risk prediction models using the top 10 variables of importance separately (the RF top 10 models and the XGBoost top 10 models mentioned in the results section) and found that the overall predictive performance of these models was higher than those constructed using only insulin-related indexes. Subsequently, we used the PDP method and SHAP method to interpret the RF top 10 models and the XGBoost top 10 models separately, further confirming the central role of HOMA-IR and TyG-WC in predicting MASLD risk. It is worth noting that in the XGBoost top 10 models, age consistently influences the occurrence and development of MASLD across the entire range.

Obviously, with increasing age, the body’s metabolism, liver function, and fat metabolism abilities weaken, particularly the decreased ability of liver cells to metabolize fat, leading to fat accumulation in the liver and thereby increasing the risk of MASLD. Additionally, as age increases, the incidence of chronic diseases such as hypertension, DM, and CVD rises. These chronic diseases have a mutually influencing relationship with MASLD, and they may worsen due to the influence of MASLD, further exacerbating the disease burden of MASLD, and forming a vicious cycle (47, 48). Furthermore, AST can reflect the degree of liver cell damage, liver inflammation, and fibrosis (49). Therefore, it plays a supplementary role in predicting MASLD risk, thereby optimizing the effectiveness of MASLD risk prediction models. Although the variables included in the two types of models differ significantly, and there are also differences in the ranking of importance, the overall predictive performance of the RF top 10 models and the XGBoost top 10 models does not differ significantly. We believe the main reason is that HOMA-IR and TyG-WC play a primary role in predicting MASLD risk, while other indicators further complement aspects not captured by these two indicators. Considering that TyG-related parameters simultaneously enter the model, there may be multicollinearity issues, leading to the model repetitively capturing the biological information or metabolic status reflected by TyG-related indicators and neglecting other potential indexes. The study also found that MASLD incidence risk varies significantly across different ethnicities, which may be attributed to differences in metabolic characteristics between ethnic groups, particularly in insulin resistance, lipid metabolism, and fat distribution. Mexican Americans tend to exhibit higher levels of insulin resistance and abdominal obesity (50), while Non-Hispanic Blacks individuals typically have a higher proportion of subcutaneous fat, with relatively less visceral fat, which may confer some protection to liver health (51). Additionally, factors such as diet and lifestyle, as well as socioeconomic status, may also contribute to the differences in MASLD incidence risk among ethnicities.

Based on the PDP and SHAP results of the RF top 10 models and the XGBoost top 10 models, we further screened the variables and constructed the optimal MASLD risk prediction model using HOMA-IR, TyG-WC, age, AST, and ethnicity. This model not only has the largest average AUC value, but also other evaluation metrics are overall better than the previously constructed model. Therefore, we believe that although insulin resistance and obesity (especially central obesity) are core factors in developing MASLD, considering other factors that reflect liver function simultaneously helps improve the effectiveness of constructing clinical prediction models for MASLD risk.

Although the predictive performance of the final models constructed in this study is superior to that of previous research, there are still some limitations. Firstly, this study relied solely on the NHANES database, which includes data from the American population, without incorporating external datasets to further validate and optimize the model’s performance. Moreover, the cross-sectional nature of the NHANES data limits the ability to establish causal relationships between variables and MASLD, which should be acknowledged as a constraint on causal inference in this study. In addition, there may be other factors affecting the prediction of MASLD risk that were not considered in this study or were not collected in the NHANES database, such as indicators of fibrosis, other indicators of inflammation, and so on, which may play a role in the diagnosis and prediction of MASLD.

## Conclusions

Our study found that HOMA-IR and TyG-WC are core factors in predicting MASLD risk. However, integrating multiple factors can further enhance the model’s predictive performance. Ultimately, our study constructed the optimal MASLD risk prediction model using HOMA-IR, TyG-WC, age, AST, and ethnicity.

## Data Availability

The raw data supporting the conclusions of this article will be made available by the authors, without undue reservation.
