# Supplementary material for: Development and validation of machine learning models for MASLD: based on multiple potential screening indicators
Source: Front Endocrinol (Lausanne). 2025 Jan 21;15:1449064. doi: 10.3389/fendo.2024.1449064 (PMC11790477; doi:10.3389/fendo.2024.1449064)
Supplement: Supplementary file 1 [file Table1.docx]

Supplementary Tables 1. Evaluation metrics for predictive performance of seven machine learning models constructed using all variables.

| indicators | XGBoost | RF | BPNN | SVM | LR | NBM | KNN | Mean |
| --- | --- | --- | --- | --- | --- | --- | --- | --- |
| Accuracy | 0.903 | 0.904 | 0.902 | 0.898 | 0.894 | 0.855 | 0.844 | 0.886 |
| Sensitivity | 0.802 | 0.755 | 0.823 | 0.705 | 0.738 | 0.743 | 0.481 | 0.721 |
| Specificity | 0.937 | 0.954 | 0.928 | 0.962 | 0.947 | 0.893 | 0.965 | 0.940 |
| FPR | 0.063 | 0.046 | 0.072 | 0.038 | 0.054 | 0.107 | 0.035 | 0.059 |
| FNR | 0.198 | 0.245 | 0.177 | 0.295 | 0.262 | 0.257 | 0.519 | 0.279 |
| PPV | 0.809 | 0.844 | 0.793 | 0.861 | 0.822 | 0.698 | 0.820 | 0.807 |
| NPV | 0.934 | 0.921 | 0.940 | 0.907 | 0.916 | 0.912 | 0.848 | 0.911 |
| F1 score | 0.805 | 0.797 | 0.807 | 0.779 | 0.778 | 0.711 | 0.606 | 0.725 |

Supplementary Tables 2. Evaluation metrics for predictive performance of seven machine learning models constructed using insulin-related indexes.

| indicators | XGBoost | RF | BPNN | SVM | LR | NBM | KNN | Mean |
| --- | --- | --- | --- | --- | --- | --- | --- | --- |
| Accuracy | 0.883 | 0.901 | 0.905 | 0.892 | 0.886 | 0.872 | 0.899 | 0.891 |
| Sensitivity | 0.760 | 0.789 | 0.806 | 0.705 | 0.658 | 0.726 | 0.747 | 0.742 |
| Specificity | 0.925 | 0.938 | 0.938 | 0.955 | 0.962 | 0.921 | 0.949 | 0.941 |
| FPR | 0.075 | 0.062 | 0.062 | 0.045 | 0.038 | 0.079 | 0.051 | 0.059 |
| FNR | 0.241 | 0.211 | 0.194 | 0.295 | 0.342 | 0.274 | 0.253 | 0.259 |
| PPV | 0.773 | 0.810 | 0.813 | 0.839 | 0.853 | 0.754 | 0.831 | 0.810 |
| NPV | 0.920 | 0.930 | 0.935 | 0.906 | 0.894 | 0.910 | 0.918 | 0.916 |
| F1 score | 0.766 | 0.799 | 0.809 | 0.766 | 0.743 | 0.740 | 0.787 | 0.773 |

Supplementary Tables 3. Evaluation metrics for predictive performance of seven machine learning models constructed using demographic characteristics variables.

| indicators | XGBoost | RF | BPNN | SVM | LR | NBM | KNN | Mean |
| --- | --- | --- | --- | --- | --- | --- | --- | --- |
| Accuracy | 0.843 | 0.856 | 0.848 | 0.855 | 0.856 | 0.835 | 0.822 | 0.845 |
| Sensitivity | 0.608 | 0.574 | 0.688 | 0.565 | 0.616 | 0.654 | 0.380 | 0.584 |
| Specificity | 0.921 | 0.951 | 0.901 | 0.952 | 0.937 | 0.896 | 0.969 | 0.932 |
| FPR | 0.079 | 0.049 | 0.052 | 0.048 | 0.063 | 0.104 | 0.031 | 0.061 |
| FNR | 0.392 | 0.426 | 0.506 | 0.435 | 0.384 | 0.346 | 0.620 | 0.444 |
| PPV | 0.720 | 0.795 | 0.700 | 0.798 | 0.764 | 0.677 | 0.804 | 0.751 |
| NPV | 0.876 | 0.870 | 0.896 | 0.868 | 0.880 | 0.886 | 0.824 | 0.871 |
| F1 score | 0.660 | 0.667 | 0.598 | 0.662 | 0.682 | 0.665 | 0.516 | 0.636 |

Supplementary Tables 4. Evaluation metrics for predictive performance of seven machine learning models constructed using other indexes.

| indicators | XGBoost | RF | BPNN | SVM | LR | NBM | KNN | Mean |
| --- | --- | --- | --- | --- | --- | --- | --- | --- |
| Accuracy | 0.788 | 0.795 | 0.819 | 0.811 | 0.812 | 0.773 | 0.812 | 0.801 |
| Sensitivity | 0.426 | 0.426 | 0.426 | 0.363 | 0.388 | 0.426 | 0.392 | 0.407 |
| Specificity | 0.909 | 0.918 | 0.951 | 0.961 | 0.954 | 0.889 | 0.952 | 0.933 |
| FPR | 0.092 | 0.082 | 0.049 | 0.044 | 0.046 | 0.111 | 0.048 | 0.067 |
| FNR | 0.574 | 0.574 | 0.574 | 0.654 | 0.612 | 0.574 | 0.608 | 0.596 |
| PPV | 0.608 | 0.635 | 0.743 | 0.754 | 0.736 | 0.561 | 0.732 | 0.681 |
| NPV | 0.826 | 0.827 | 0.832 | 0.819 | 0.824 | 0.823 | 0.824 | 0.825 |
| F1 score | 0.501 | 0.542 | 0.516 | 0.490 | 0.508 | 0.484 | 0.511 | 0.507 |

Supplementary Tables 5. Evaluation metrics for predictive performance of seven machine learning models constructed using the top 10 variables of importance in the RF model.

| indicators | XGBoost | RF | BPNN | SVM | LR | NBM | KNN | Mean |
| --- | --- | --- | --- | --- | --- | --- | --- | --- |
| Accuracy | 0.913 | 0.913 | 0.921 | 0.908 | 0.905 | 0.864 | 0.880 | 0.901 |
| Sensitivity | 0.827 | 0.806 | 0.861 | 0.747 | 0.726 | 0.730 | 0.696 | 0.770 |
| Specificity | 0.942 | 0.949 | 0.941 | 0.962 | 0.965 | 0.909 | 0.941 | 0.944 |
| FPR | 0.058 | 0.051 | 0.059 | 0.038 | 0.035 | 0.092 | 0.059 | 0.056 |
| FNR | 0.173 | 0.194 | 0.139 | 0.253 | 0.274 | 0.270 | 0.304 | 0.230 |
| PPV | 0.827 | 0.841 | 0.829 | 0.868 | 0.873 | 0.727 | 0.797 | 0.823 |
| NPV | 0.942 | 0.936 | 0.953 | 0.919 | 0.913 | 0.910 | 0.903 | 0.925 |
| F1 score | 0.827 | 0.823 | 0.845 | 0.803 | 0.793 | 0.728 | 0.743 | 0.794 |

Supplementary Tables 6. Evaluation metrics for predictive performance of seven machine learning models constructed using the top 10 variables of importance in the XGBoost model.

| indicators | XGBoost | RF | BPNN | SVM | LR | NBM | KNN | Mean |
| --- | --- | --- | --- | --- | --- | --- | --- | --- |
| Accuracy | 0.913 | 0.916 | 0.904 | 0.907 | 0.905 | 0.873 | 0.883 | 0.900 |
| Sensitivity | 0.823 | 0.814 | 0.781 | 0.755 | 0.734 | 0.662 | 0.549 | 0.731 |
| Specificity | 0.944 | 0.951 | 0.945 | 0.958 | 0.962 | 0.944 | 0.968 | 0.953 |
| FPR | 0.056 | 0.049 | 0.055 | 0.042 | 0.038 | 0.056 | 0.032 | 0.047 |
| FNR | 0.170 | 0.186 | 0.219 | 0.245 | 0.266 | 0.338 | 0.451 | 0.268 |
| PPV | 0.830 | 0.847 | 0.826 | 0.857 | 0.866 | 0.797 | 0.850 | 0.839 |
| NPV | 0.940 | 0.939 | 0.928 | 0.921 | 0.916 | 0.893 | 0.865 | 0.915 |
| F1 score | 0.830 | 0.830 | 0.803 | 0.803 | 0.796 | 0.724 | 0.667 | 0.779 |

Supplementary Tables 7. Evaluation metrics for predictive performance of seven machine learning models constructed using HOMA-IR, TyG-WC, age, AST, ethnicity.

| indicators | XGBoost | RF | BPNN | SVM | LR | NBM | KNN | Mean |
| --- | --- | --- | --- | --- | --- | --- | --- | --- |
| Accuracy | 0.918 | 0.905 | 0.912 | 0.910 | 0.907 | 0.877 | 0.904 | 0.905 |
| Sensitivity | 0.835 | 0.798 | 0.848 | 0.755 | 0.722 | 0.654 | 0.751 | 0.780 |
| Specificity | 0.945 | 0.941 | 0.934 | 0.962 | 0.961 | 0.951 | 0.955 | 0.950 |
| FPR | 0.055 | 0.059 | 0.066 | 0.038 | 0.039 | 0.049 | 0.045 | 0.050 |
| FNR | 0.165 | 0.203 | 0.152 | 0.244 | 0.278 | 0.346 | 0.249 | 0.234 |
| PPV | 0.835 | 0.818 | 0.811 | 0.869 | 0.859 | 0.816 | 0.848 | 0.837 |
| NPV | 0.945 | 0.933 | 0.949 | 0.922 | 0.912 | 0.892 | 0.920 | 0.925 |
| F1 score | 0.835 | 0.808 | 0.829 | 0.808 | 0.784 | 0.726 | 0.796 | 0.798 |


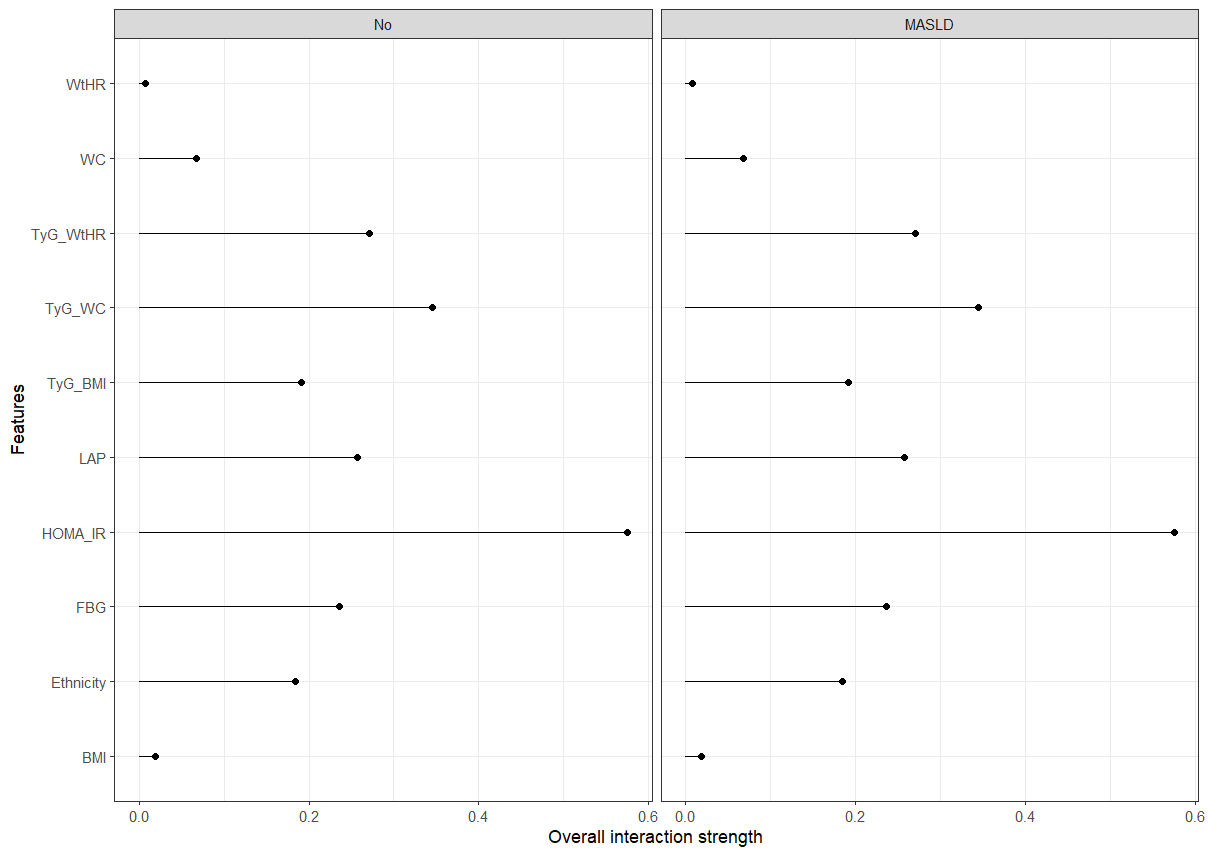


Supplementary Figure 1. The RF top 10 variables’ strength of interactions


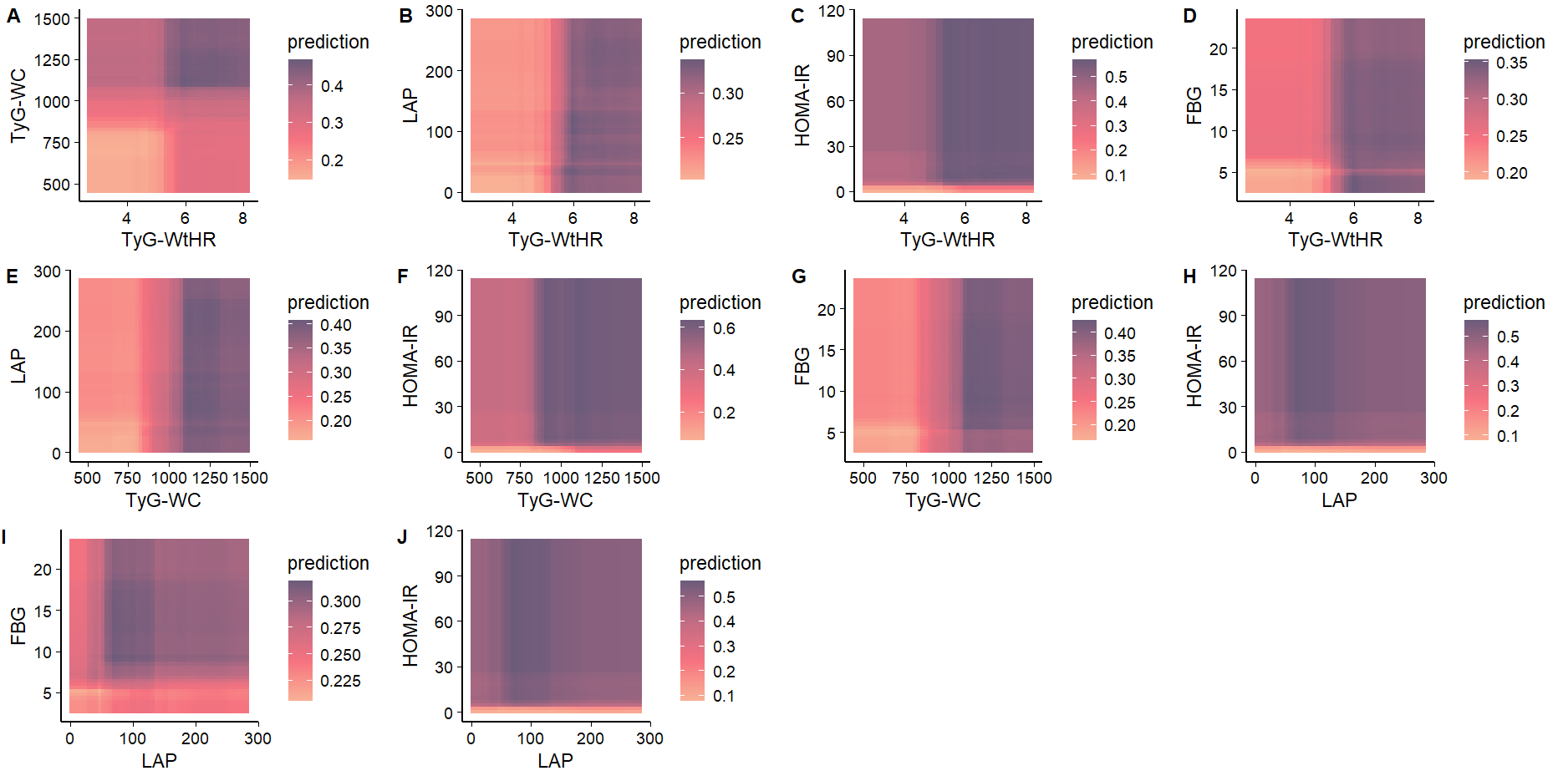


Supplementary Figure 2. Synergistic effects between the RF top 10 variables on MASLD.


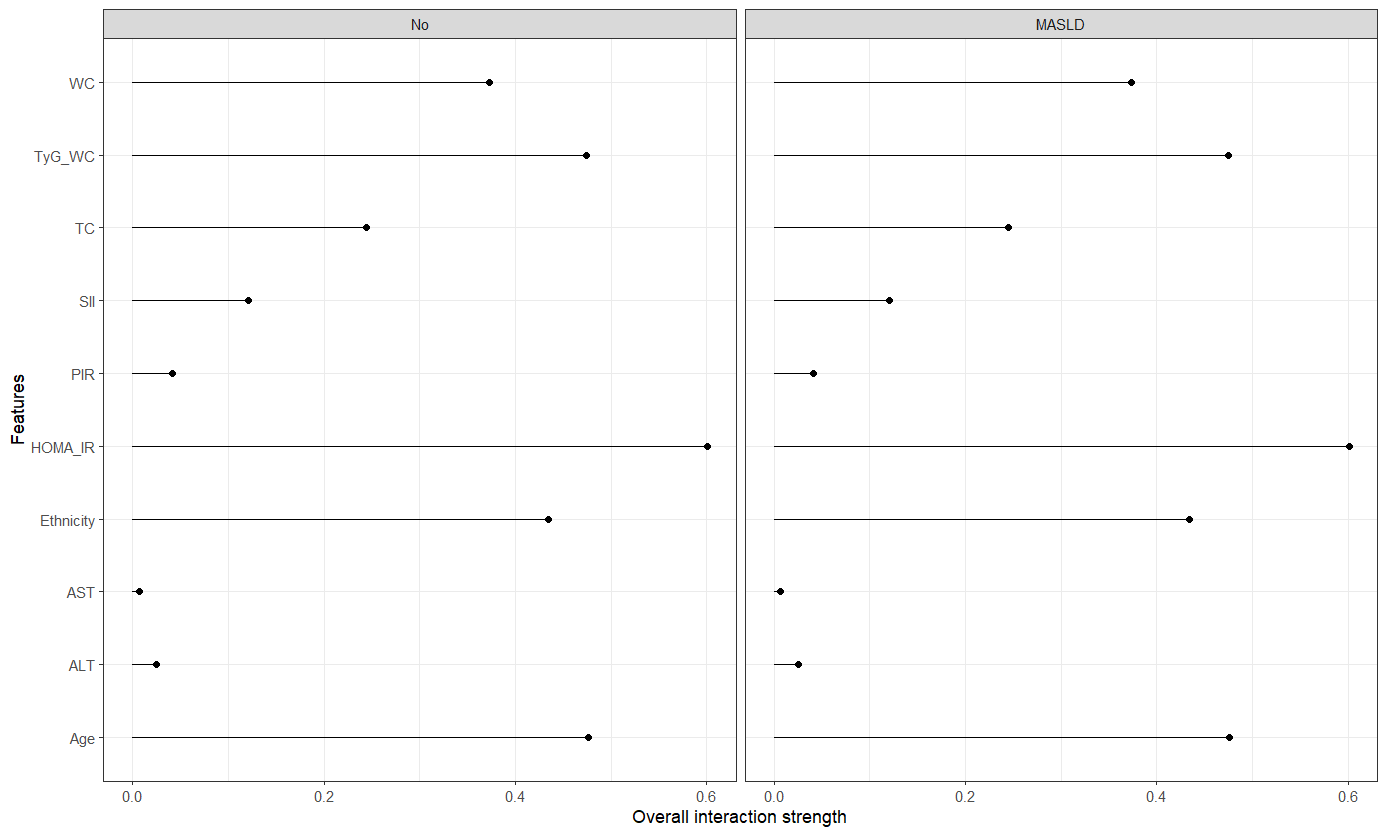


Supplementary Figure 3. The XGBoost top 10 variables’ strength of interactions


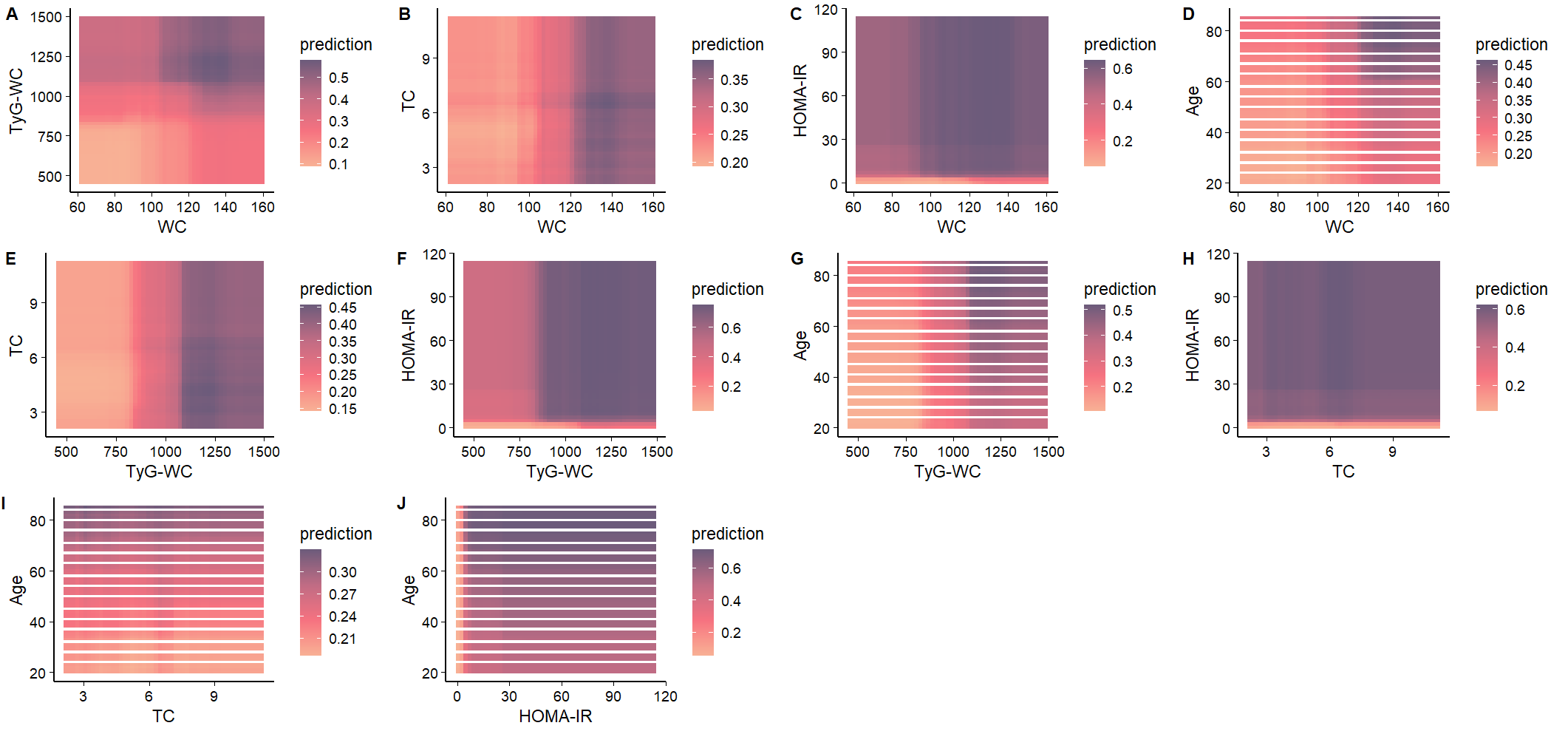


Supplementary Figure 4. Synergistic effects between the XGBoost top 10 variables on MASLD.


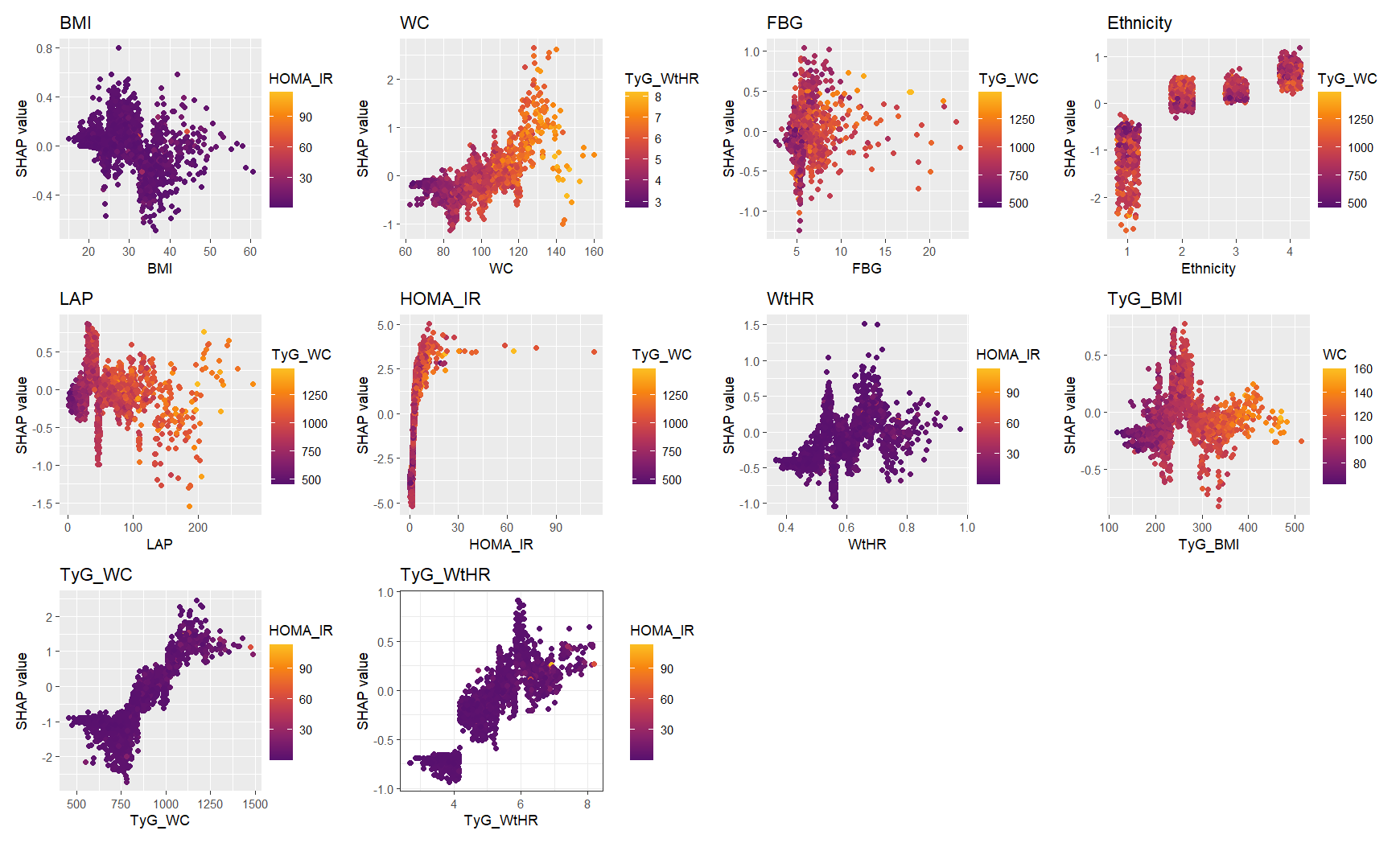


Supplementary Figure 5.SHAP dependence plot of the RF top 10 models


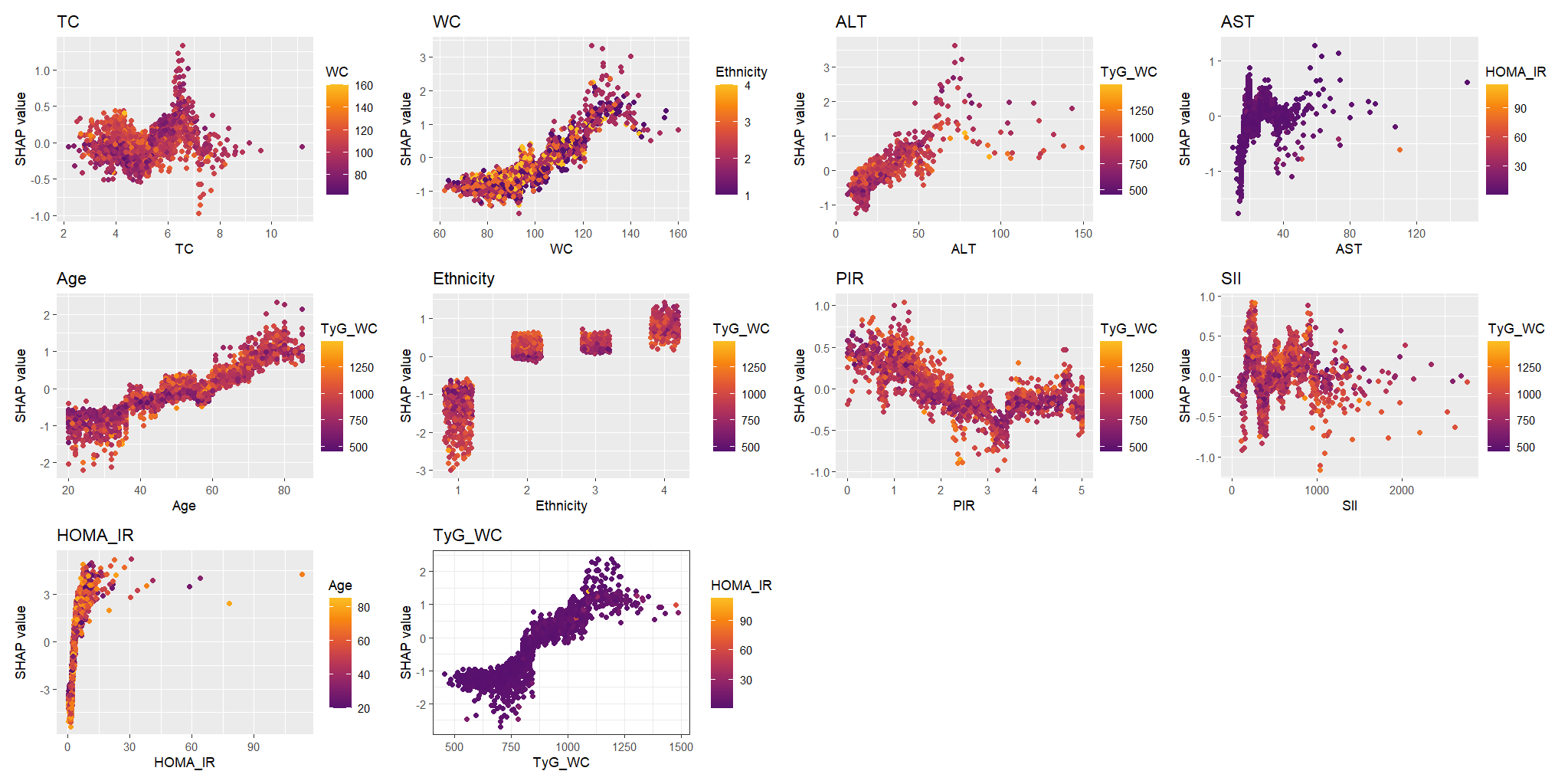


Supplementary Figure 6.SHAP dependence plot of the XGBoost top 10 models
